# Network‐based identification of key proteins and repositioning of drugs for non‐small cell lung cancer

**DOI:** 10.1002/cnr2.2031

**Published:** 2024-04-10

**Authors:** Oluwatosin Maryam Adeyemo, Zainab Ashimiyu‐Abdusalam, Mary Adewunmi, Temitope Ayanfunke Ayano, Muhammad Sohaib, Reem Abdel‐Salam

**Affiliations:** ^1^ Department of Biochemistry Federal University of Technology Akure Nigeria; ^2^ Cancer Research with AI (CaresAI) Hobart Australia; ^3^ Department of Biochemistry and Nutrition Nigeria Institute of Medical Research Lagos Nigeria; ^4^ College of Health and Medicine University of Tasmania Hobart Tasmania Australia; ^5^ Department of Microbiology Obafemi Awolowo University Ile‐Ife Nigeria; ^6^ Department of Computer Engineering, Faculty of Engineering Cairo University Cairo Egypt

**Keywords:** drug repurposing, drug–gene interaction, network analysis, non‐small cell lung cancer (NSCLC), therapeutics

## Abstract

**Background:**

NSCLC is a lethal cancer that is highly prevalent and accounts for 85% of cases of lung cancer. Conventional cancer treatments, such as chemotherapy and radiation, frequently exhibit limited efficacy and notable adverse reactions. Therefore, a drug repurposing method is proposed for effective NSCLC treatment.

**Aims:**

This study aims to evaluate candidate drugs that are effective for NSCLC at the clinical level using a systems biology and network analysis approach.

**Methods:**

Differentially expressed genes in transcriptomics data were identified using the systems biology and network analysis approaches. A network of gene co‐expression was developed with the aim of detecting two modules of gene co‐expression. Following that, the Drug–Gene Interaction Database was used to find possible drugs that target important genes within two gene co‐expression modules linked to non‐small cell lung cancer (NSCLC). The use of Cytoscape facilitated the creation of a drug–gene interaction network. Finally, gene set enrichment analysis was done to validate candidate drugs.

**Results:**

Unlike previous research on repositioning drugs for NSCLC, which uses a gene co‐expression network, this project is the first to research both gene co‐expression and co‐occurrence networks. And the co‐occurrence network also accounts for differentially expressed genes in cancer cells and their adjacent normal cells. For effective management of non‐small cell lung cancer (NSCLC), drugs that show higher gene regulation and gene affinity within the drug–gene interaction network are thought to be important. According to the discourse, NSCLC genes have a lot of control over medicines like vincristine, fluorouracil, methotrexate, clotrimazole, etoposide, tamoxifen, sorafenib, doxorubicin, and pazopanib.

**Conclusion:**

Hence, there is a possibility of repurposing these drugs for the treatment of non‐small‐cell lung cancer.

## 1 INTRODUCTION

According to GLOBOCAN 2020, lung cancer has the highest mortality rate^1^ and is responsible for 18% of the deaths associated with cancer worldwide.^2^ Nearly 84% of lung cancer cases are non‐small cell lung carcinoma (NSCLC) and 15% are small cell lung carcinoma (SCLC).^3^ NSCLC is categorized into three sub‐types: adenocarcinoma, squamous cell carcinoma, and large cell carcinoma. Approximately 45% of all non‐small cell lung cancers are adenocarcinomas, followed by 25%–30% of squamous cell carcinomas and 5%–10% of large cell carcinomas.^3^ The poor survival rate of lung cancer patients at the metastatic stage is primarily attributable to the late diagnosis of disease at stages III and IV.^4^ Approximately 92% of patients diagnosed at stage IA1 could live for at least 5 years,^5^ compared to 10% of those diagnosed at stage IV. In addition, a slight increase in tumor size from 1 cm (stage IA1) to >2 cm (stage IA3) could reduce patients' 5‐year survival rates from 92% to 77%.^5^ Current treatments for non‐small cell lung cancer, including surgery, chemotherapy, and radiotherapy, are insufficient to reduce the high mortality rates.^6^, ^7^ Additionally, the non‐specificity of chemotherapeutics leads to serious adverse effects that harm healthy cells.^8^ To improve the survival rate of NSCLC patients, individualized treatment is preferred. Epidermal growth factor receptor tyrosine kinase inhibitors (EGFR‐TKIs), a new type of molecularly targeted therapy, may stop lung tumors with EGFR mutations from growing and spreading. LC is a diverse disease with complicated molecular mechanisms for uncontrolled cell growth. These mechanisms could be caused by promoter methylation, dysregulated gene expression, and/or mutations in genes that stop tumors from growing and genes that make cancer cells grow.^9^ In this context, it is imperative to gain a comprehensive understanding of the underlying mechanism of action in order to ascertain an effective strategy for addressing the ailment.

Drug repositioning, also known as drug repurposing, serves as a cost‐effective and time‐efficient approach to uncovering new drugs.^10^ Typically, there are three primary strategies for drug repurposing, which encompass experimental biological techniques, computational approaches, and a combination of both.^11^ Of the computational approaches, network‐based methods play an essential role in drug repositioning. Previous studies have used this method to discover drugs that can be repurposed for different diseases. It was found that mefloquine, an FDA‐approved drug used to treat malaria, could also be used to treat cancer by targeting the A2AR (Adenosine 2A Receptor) gene in breast and prostate cancer.^12^ Similarly, Wang et al.^13^ used weighted gene co‐expression network analysis (WGCNA) to identify 15 drugs with potential for treating melanoma. Crizotinib, an ALK inhibitor that is used to treat NSCLC, was also found to have potential as a treatment for AML and multiple myeloma cells.^14^ In the present study, we employ a computational drug repositioning approach to pinpoint potential drugs for the treatment of NSCLC by using network analysis and protein interaction. The experiment looks at gene co‐expression and co‐occurrence networks. It is different from earlier research on NSCLC drug repositioning, which used a gene co‐expression network.^15^ In addition, the co‐occurrence network considers the differential expression of genes between cancer cells and their adjacent normal cells. This study aims to evaluate candidate drugs that are effective for NSCLC at the clinical level using a systems biology and network analysis approach.

## 2 METHODOLOGY

### 2.1 Dataset and preprocessing

The gene expression data from the NCBI Gene Expression Omnibus (GEO) database was utilized, with reference to the corresponding accession numbers. GSE27262^16^, ^17^ and GSE21933^18^ to compare non‐small cell lung cancer (NSCLC) and normal cell transcriptomes. The accession numbers of the platforms used are GPL570 and GPL6254, respectively. The first sample (GSE27262) contains 50 samples, with 25 normal and 25 non‐small cell lung cancer. The average age of the sample is 58, with a standard deviation of 12.5. The second sample (GSE21933) contains 42 samples with 21 normal and 21 non‐small cell lung cancer, with an average age of 70 and a 7.8 standard deviation. The GEO2R program was used to find differentially expressed genes (DEGs) between these two groups.^19^ A significance level of *p*‐value <.05 and Log2Fold >2.0 were used to narrow down the list of differentially expressed genes. A Venn diagram (http://bioinformatics.psb.ugent.be/webtools/Venn/) was used to visualize the intercepting genes in the two datasets. This rigorous selection process allowed us to focus on the most robust and biologically relevant genes for subsequent analyses.

### 2.2 Network construction

It was possible to find interactions between differentially expressed genes (DEGs) in the STRING database for humans. This helped us learn more about how non‐small cell lung cancer (NSCLC) works.^20^ Using a minimum interaction score of 0.04 as a starting point, a thorough human protein interactome network and a gene co‐expression and co‐occurrence network were both created. The co‐expression analysis in STRING uses a method known as FAVA (Functional Associations using Variational Autoencoders).^21^ This network was then transferred to the Cytoscape software^22^ for further analysis. To find areas of the network that are strongly connected, we used the Molecular Complex Detection (MCODE) algorithm,^23^ which is a graph‐theoretic clustering method designed to do just that. Based on the cluster's and the neighborhood's densities, this algorithm locates seed nodes and grows them.^23^ We utilized the MCODE algorithm with the following parameters: degree threshold = 2, node score threshold = 0.2, K‐core threshold = 2, and maxdepth = 100. Using the MCODE algorithm, we were able to find two separate gene co‐expression and co‐occurrence modules in the network. These modules represent biologically relevant regions within the network that may have functional significance in NSCLC tumorigenesis. Using the MCODE algorithm helped us find these modules in a thorough and accurate way. This gave us a better understanding of how differentially expressed genes and possible molecular pathways involved in NSCLC work together.

### 2.3 Enrichment analysis

Gene co‐expression and co‐occurrence analysis helped us find the largest component network. We then used Metascape^24^ for functional enrichment analysis to learn more about the possible biological functions and pathways linked to it. Metascape is a powerful tool that enables us to explore the biological functions and pathways of our gene sets in a comprehensive and intuitive manner.^25^ We narrowed down our search using six words: GO‐BP (Gene Ontology Biological Process), KEGG (Kyoto Encyclopedia of Genes and Genomes) pathways, Reactome pathways, WikiPathways, Canonical pathways, and CORUM pathway.^25^ These terms were selected for their relevance to NSCLC and their potential to illuminate molecular pathways implicated in carcinogenesis. We also used Metascape's Molecular Complex Detection (MCODE) method to group the enriched phrases into bigger groups so that we could find biological themes that were common and learn more about how NSCLC works. Clustering these terms revealed NSCLC tumors' most enriched and significant biological processes and pathways.

### 2.4 Drug–gene interaction

It was the Drug Gene Interaction Database (DGIdb) that helped us find drugs that might work on key genes in the two sets of genes that we found together.^26^ The DGIdb is a comprehensive database that integrates multiple sources of information to identify potential drug targets and interactions. Using this database, we were able to identify candidate drugs that target key genes within the two modules, providing us with a list of potential drug candidates for further investigation. We made a drug–gene interaction network using Cytoscape, which is powerful software for seeing and analyzing complex networks. This helped us learn more about how these medicines might interact with the important genes in the modules.^22^ The drug–gene interaction network allowed us to gain a more comprehensive understanding of the potential interactions between the candidate drugs and the key genes within the modules, highlighting potential drug targets.

### 2.5 Candidate drug validation

We used the STITCH database and gene set enrichment analysis (GSEA) to further validate the probable candidate medications discovered through our investigation. With the help of the sophisticated computational technique GSEA, we can identify whether a group of genes exhibits statistically significant variations between two biological states.^27^ In this case, we used GSEA to check if the chosen candidate drugs were high in NSCLC‐related gene sets,^27^ which gave us information about how well they might work for treating NSCLC. In addition to GSEA, we also utilized the STITCH database, a comprehensive resource that integrates various sources of data to identify potential drug targets and interactions.^28^ By using the STITCH database, we were able to evaluate the identified candidate drugs and determine whether they had previously been shown to be associated with NSCLC or related biological pathways.

## 3 RESULTS

### 3.1 Differentially expressed genes analysis

**FIGURE 1:**
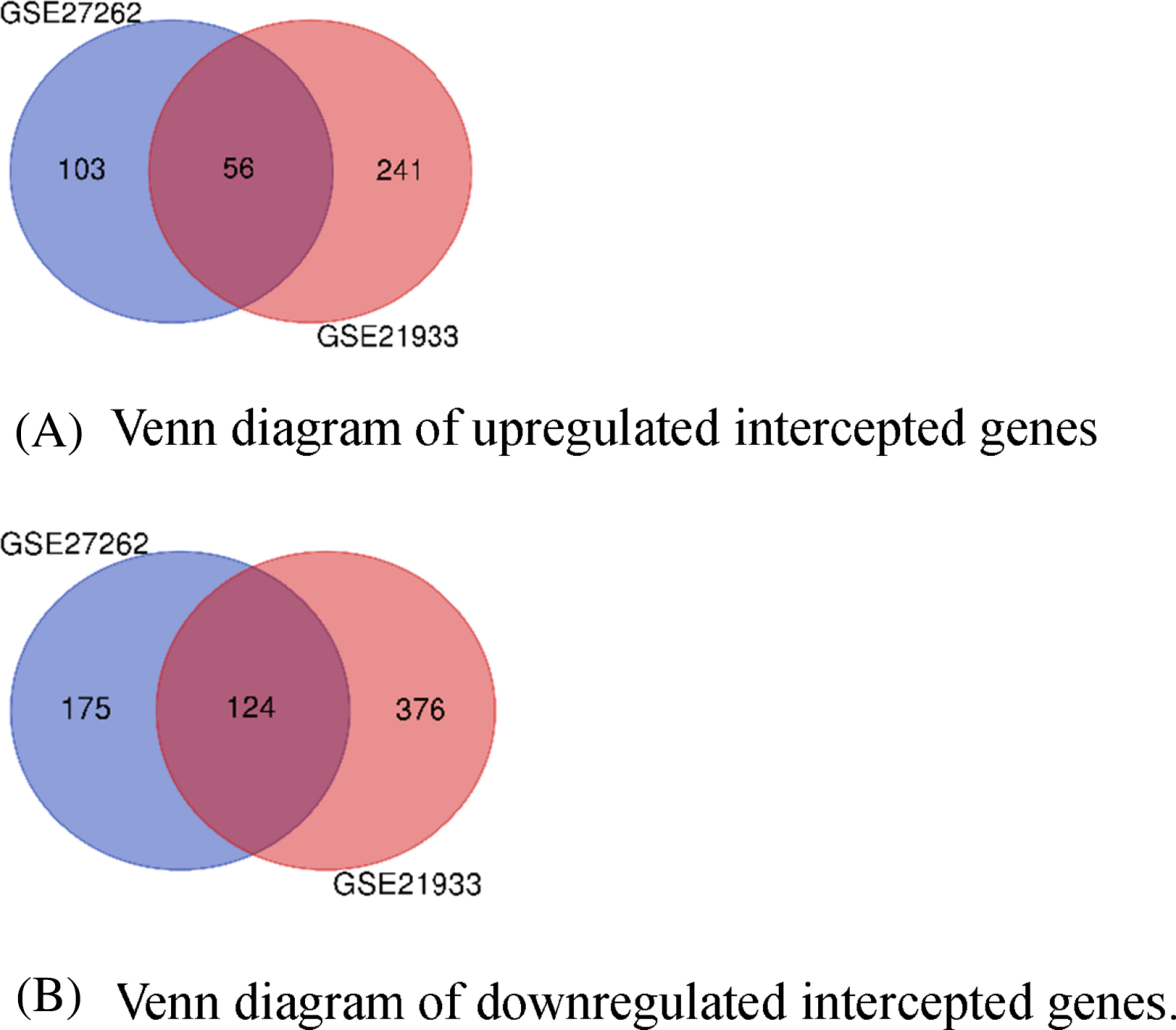
(A) Venn diagram of upregulated intercepted genes. (B) Venn diagram of downregulated intercepted genes.

According to gene expression data from two independent studies, GSE27262 and GSE21933, a total of 50 and 42 samples were analyzed, respectively. Utilizing the GEO2R tool, it was found that 458 genes exhibited differential expression in GSE27262, while GSE21933 displayed differential expression in 797 genes. Among these genes, a total of 400 were upregulated, indicating increased expression levels, while 675 genes were downregulated, indicating decreased expression levels (Figure 1). The relevant genes were used for subsequent analysis.

### 3.2 Module analysis and gene co‐expression network construction

A gene co‐expression and co‐occurrence network was carefully built using the STRING database to look into the relationships and interactions between genes that were differentially expressed in both the GSE27262 and GSE21933 datasets. Prior to network construction, any disconnected genes were systematically removed from the analysis, ensuring a focused and interconnected network. As a result, the resulting network encompassed a substantial number of nodes, totaling 1024, and demonstrated numerous edges, amounting to 8652 in total.

**FIGURE 2:**
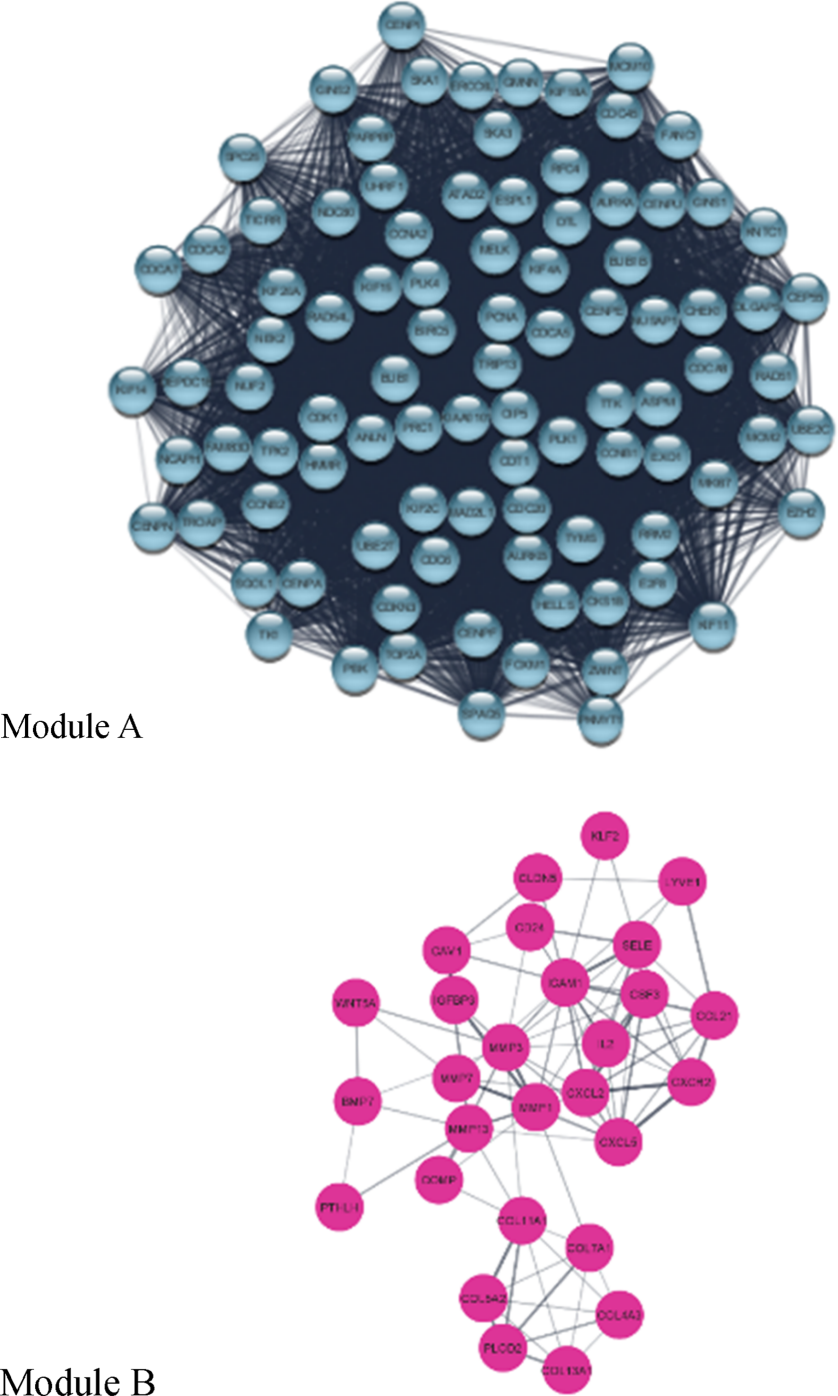
Modules (A and B) of the interaction network of connected genes drawn using Cytoscape^29^ v. 3.9.1. The circle nodes represent genes, while the line edges show their interactions.

To delve deeper into the NSCLC human interactome data, the gene co‐expression network was further analyzed using the STRING database. The MCODE algorithm was used to successfully find two separate gene modules (Figure 2) in the human interactome network using this method. These modules effectively represented clusters of genes that displayed significant co‐expression patterns and shared functional relationships. Notably, the purple module emerged as the smaller module, housing 28 genes that exhibited intricate interconnections and concerted expression behavior. On the other hand, the largest module, known as the blue module, comprised an impressive collection of 92 genes, showcasing a robust network of interdependencies and interplay.

### 3.3 Enrichment analysis of gene modules

**FIGURE 3:**
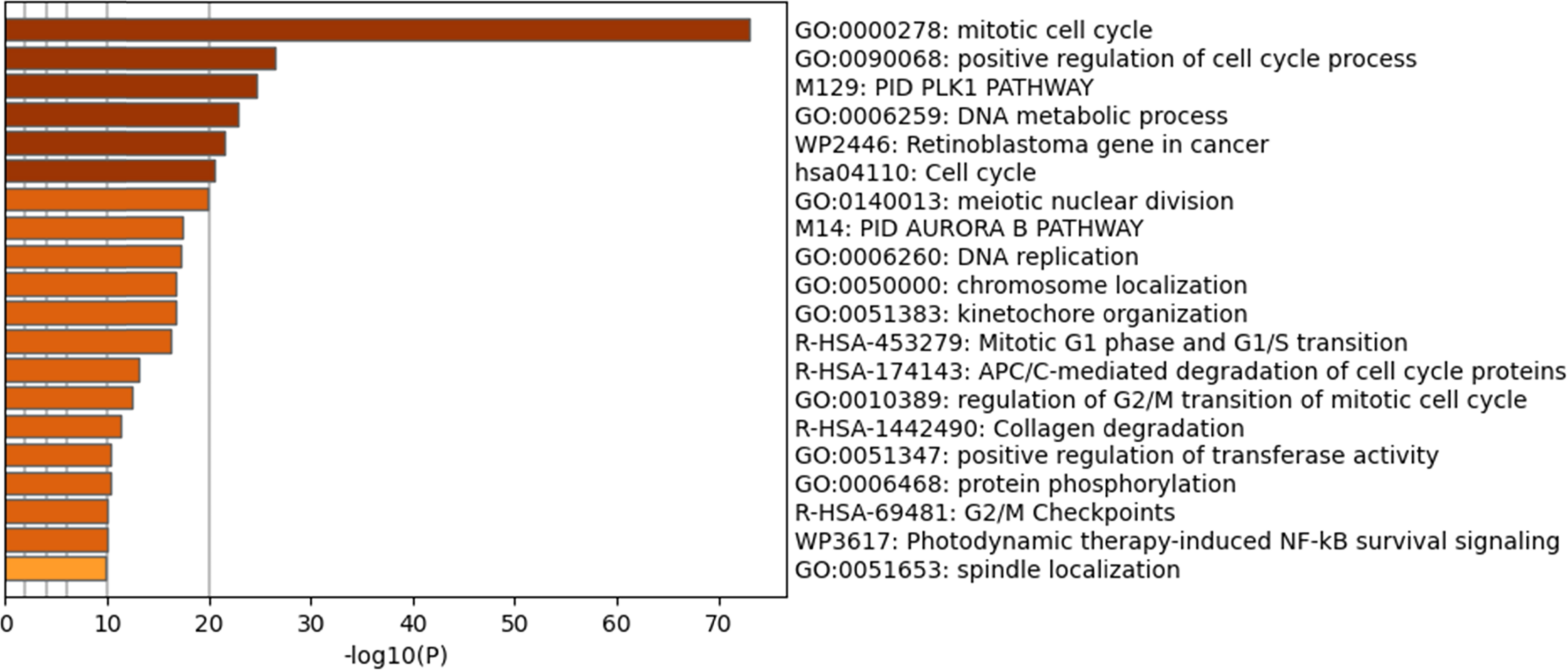
Bar graph of *p*‐value‐colored enriched terms from gene lists obtained from Metascape.^24^

The biological function of the genes in the two modules was carried out using a hypergeometric test and the Benjamini‐Hochberg statistical correction algorithm in Metascape. It was done using the following ontology sources: KEGG Pathway,^30^ Reactome Gene Sets,^31^ CORUM,^32^ PANTHER Pathway,^33^ and WikiPathways.^34^ The result shows that the terms were primarily involved in the mitotic cell cycle, kinetochore organization, and DNA metabolic processes (Figure 3). They are also involved in the degradation of collagen and cell cycle proteins.

**FIGURE 4:**
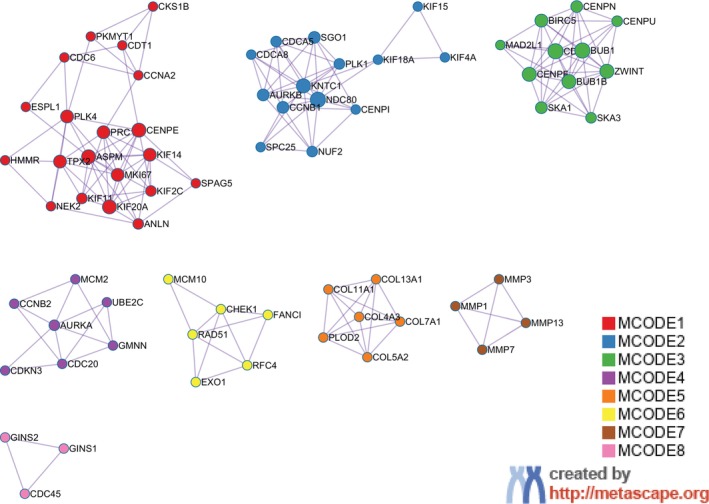
Module detection using the MCODE algorithm in Metascape.^24^ Circles represent protein nodes. Nodes in each subgraph are colored differently for different modules.

In addition, clustering analysis was done using the MCODE algorithm in Metascape, resulting in eight modules (Figure 4), for molecular detection and to identify similar biological themes and relevant biological pathways associated with NSCLC. As detailed in Table 1, the genes are associated with the cell cycle and involved in processes such as mitosis, collogen formation, DNA replication, and repair.

**TABLE 1:** Clustering of functional enrichment analysis.

Functional component	Term ID	Biological function	Log 10 (*p*‐value)
MCODE_1	GO:0051301	Cell division	−24.8
	GO:1903047	Mitotic cell cycle process	−24.7
	GO:0000278	Mitotic cell cycle	−23.5
MCODE_2	R‐HSA‐2500257	Resolution of Sister Chromatid Cohesion	−26.8
	R‐HSA‐68877	Mitotic Prometaphase	−24.2
	R‐HSA‐68882	Mitotic Anaphase	−23.5
MCODE_3	R‐HSA‐2500257	Resolution of Sister Chromatid Cohesion	−22.9
	R‐HSA‐141444	Amplification of signal from unattached kinetochores via a MAD2 inhibitory signal	−20.9
	R‐HSA‐141424	Amplification of signal from the kinetochores	−20.9
MCODE_4	GO:1903047	Mitotic cell cycle process	−9.8
	R‐HSA‐69278	Cell Cycle, Mitotic	−9.6
	GO:0000278	Mitotic cell cycle	−9.3
MCODE_5	R‐HSA‐1650814	Collagen biosynthesis and modifying enzymes	−16.0
	R‐HSA‐1474290	Collagen formation	−15.2
	M3005	NABA COLLAGENS	−13.5
MCODE_6	WP4946	DNA repair pathways, full network	−11.3
	R‐HSA‐5693616	Presynaptic phase of homologous DNA pairing and strand exchange	−10.4
	R‐HSA‐5693579	Homologous DNA Pairing and Strand Exchange	−10.3
MCODE_7	WP129	Matrix metalloproteinases	−12.1
	R‐HSA‐1592389	Activation of Matrix Metalloproteinases	−11.9
	GO:0030574	Collagen catabolic process	−11.5
MCODE_8	R‐HSA‐176974	Unwinding of DNA	−10.3
	GO:0006268	DNA unwinding involved in DNA replication	−9.5
	R‐HSA‐69190	DNA strand elongation	−9.0

### 3.4 Drug–gene interaction and validation

**FIGURE 5:**
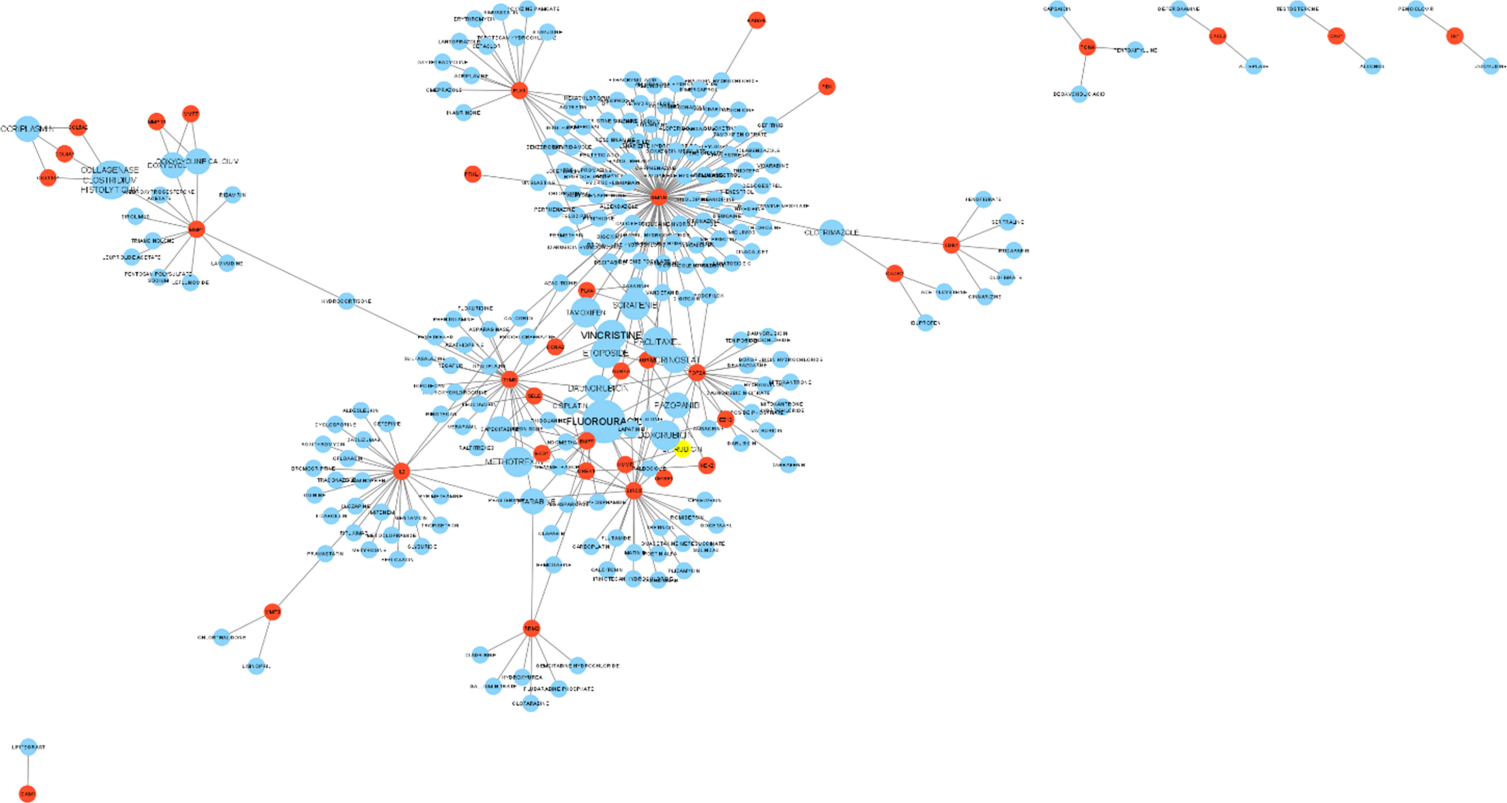
Drug–gene interaction network drawn with Cytoscape^29^ v. 3.9.1. The red nodes indicate the gene, while the blue nodes are drugs that interact with those genes. The size of the blue nodes (drug) indicates how many genes they interact with; the larger the size, the more genes they interact with.

To find possible drugs to treat NSCLC, a drug–gene interaction network was built using drugs in the Drug Gene Interaction Database (DGIdb)^26^ and genes that were co‐expressed in two important gene modules. The interaction network was reconstructed using Cytoscape^22^ v. 3.9.1. The network is shown in Figure 5. Eight drugs show high (above 3) interactions with genes, while 10 drugs have 3 interactions with genes (Table 2).

**TABLE 2:** Potential drugs with three or more target genes.

Drug	Number of interacting nodes (genes)	Target gene
Fluorouracil	8	TOP2A, AURKA, SELE, TYMS, HMMR, BIRC5, CHEK1, EXO1
Methotrexate	5	IL2, TYMS, BIRC5, BMP7, HMMR
Etoposide	5	CHEK1, TYMS, GMNN, TOP2A, AURKB
Vincristine	5	TYMS, GMNN, BIRC5, BMPT, TOP2A
Tamoxifen	4	TYMS, CCNA2, AURKA, AURKB
Sorafenib	4	AURKA, GMNN, PLK4, AURKB
Doxorubicin	4	EZH2, HMMR, BIRC5, BMPT
Collagenase Clostridium Histolyticum	4	COL11A1, COL4A3, COL5A2, MMP1
Ocriplasmin	3	COL11A1, COL4A3, COL5A2
Doxycycline	3	MMP13, MMP7, MMP1
Doxycycline calcium	3	MMP13, MMP7, MMP1
Cisplastin	3	CHEK1, TYMS, AURKA
Daunorubicin	3	TYMS, TOP2A, BMP7
Paclitaxel	3	GMNN, AURKA, EZH2
Vorinostat	3	GMNN, EZH2, BIRC5
Pazopanib	3	NEK2, AURKA, CCNA2
Clotrimazole	3	GMNN, CXCR2, CDK1
Capecitabin	3	TYMS, SELE, EXO1
Cytarabine	3	RRM2, BMP7, TYMS

**FIGURE 6:**
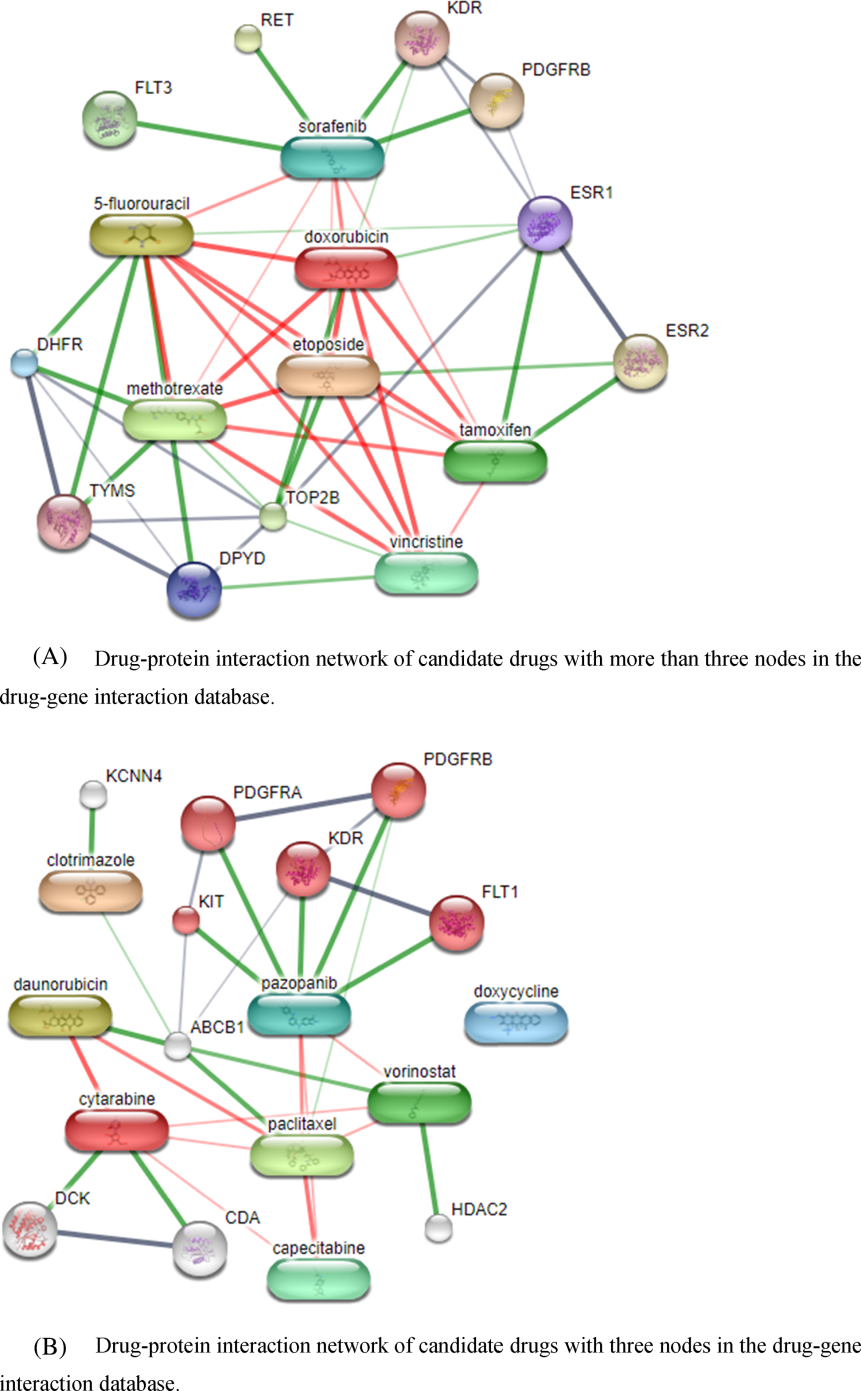
The STITCH (Kuhn et al., 2007) v5.0 network of drug–protein interactions for possible drugs that target key proteins. (A) Drug–protein interaction network in drugs with more than three nodes in the drug–gene interaction database. (B) A drug–protein interaction network of candidate drugs with three nodes in the drug–gene interaction database. ABCB1, ATP‐binding cassette subfamily B member 1; CDA, cytidine deaminase; DCK, deoxycytidine kinase; DHFR, dihydrofolate reductase; DPYD, dihydropyrimidine dehydrogenase; ESR1, estrogen receptor 1; ESR2, estrogen receptor 2; FLT1, fms‐related receptor tyrosine kinase 1; FLT3, fms‐related receptor tyrosine kinase 3; HDAC2, histone deacetylase 2; KCNN4, potassium calcium‐activated channel subfamily N member 4; KDR, kinase insert domain receptor; KIT, KIT proto‐oncogene; PDGFRA, platelet‐derived growth factor receptor alpha; PDGFRB, platelet‐derived growth factor receptor beta; RET, ret proto‐oncogene; TOP2B, DNA topoisomerase II beta; TYMS, thymidylate synthetase.

STITCH v5.0,^35^ a drug–protein interaction database, was used to validate the results from the drug–gene interaction network using a medium confidence cutoff of 0.4. The STITCH result is shown in Figure 6. Pathway enrichment analysis from STITCH shows that Sorafenib, Pazopanib, and Methotrexate interact with proteins in the peptidyl‐tyrosine phosphorylation pathway, vascular endothelial growth factor signaling pathway and uracil metabolic process and they are involved in transmembrane receptor protein tyrosine kinase activity.

## 4 DISCUSSION

The goal of this study was to discover potentially useful drugs for the treatment of non‐small cell lung cancer (NSCLC) by using a systems biology and network analysis technique to identify important genes and proteins involved in NSCLC and to build a drug–gene interaction network for potential drug repurposing. Due to the ineffectiveness and considerable side effects of conventional cancer treatments, drug repurposing has emerged as a possible method for developing new medicines for NSCLC. The study used transcriptomics data from two GEO datasets to find differentially expressed genes and build a gene co‐expression network in order to find new medication candidates. As a result, two gene co‐expression modules were discovered. Candidate medications that target crucial genes in the two NSCLC modules were found using the drug–gene interaction database. Cytoscape made it simpler to create a network of drug–gene interactions, and gene set enrichment analysis validated potential medications.

Enrichment analysis is a crucial tool for identifying significant biological processes and pathways in cancer research.^36^ In this study, the enrichment analysis revealed that several processes, including cell division, the mitotic cell cycle, collagen formation, and DNA repair, are important in cancer development and progression. Dysregulation of cell division and the mitotic cell cycle leads to uncontrolled proliferation, which is a hallmark of cancer development.^37^ The enrichment analysis showed that genes involved in the mitotic cell cycle are significantly enriched in cancer cells, highlighting their critical role in cancer development. A problem with how collagen is made and broken down was found to be very important in the spread and progression of cancer.^38^ Collagen is an important part of the extracellular matrix that gives cells support. A lot of genes that work with DNA metabolism were found to be overexpressed in cancer cells. This showed that DNA repair is badly controlled in cancer, which stops mutations from building up and causing cancer.^39^ Therefore, targeting these processes through drug intervention could offer potential therapeutic benefits for NSCLC patients.

The drug–gene interaction network analysis in this study identified seven modal genes: DNA topoisomerase II Alpha (TOP2A), Thymidylate Synthetase (TYMS), Survivin (BIRC5), Geminin DNA Replication Inhibitor (GMNN), Cyclin A2 (CCNA2), Aurora Kinase A (AURKA), and Aurora Kinase B (AURKB) that have potential roles in NSCLC treatment. These genes are known to be involved in cell division, DNA replication, and DNA repair processes, which are essential for cancer cell growth and proliferation. TOP2A is an enzyme involved in DNA replication and transcription, and its overexpression has been associated with a poor prognosis in NSCLC patients.^39^, ^40^ In addition, several TOP2A inhibitors, such as etoposide and doxorubicin, have shown promising results in preclinical studies for cancer treatment.^41^ TYMS is an important enzyme for making DNA. It has been found to be upregulated in NSCLC and could be a target for chemotherapy drugs like 5‐fluorouracil.^42^

BIRC5 (survivin) is a protein involved in cell division and is associated with drug resistance and a poor prognosis.^44^ GMNN encodes geminin, a protein that plays a critical role in DNA replication and cell cycle regulation. GMNN overexpression has been observed in breast cancer, and it is associated with a poor prognosis.^43^ CCNA2 encodes cyclin A2, a protein that plays a critical role in cell cycle regulation. In a study by Qian et al.,^45^ it was found that the overexpression of the CCNB2 protein is associated with clinical progression and a poor prognosis in NSCLC. AURKA and AURKB encode aurora kinases A and B, respectively, which play critical roles in mitosis and cell division. Overexpression of AURKA and AURKB has been observed in various cancers, including NSCLC, and is associated with poor prognosis and drug resistance.^46^ In summary, all the modal genes in these studies have potential roles in NSCLC treatment and have been previously reported as potential targets for NSCLC treatment. Previous research has found a number of drugs that could be used to treat NSCLC. These include vincristine, fluorouracil, methotrexate, clotrimazole, etoposide, tamoxifen, sorafenib, doxorubicin, and pazopanib. These drugs are good at controlling genes, so the results of this study back them up.

The gene co‐expression and co‐occurrence network approach used in this study has a unique feature that sets it apart from previous studies. The accuracy of the research results improves since it takes into consideration the variable gene expression patterns between cancer cells and the adjacent normal cells. Previous research has found a number of drugs that could be used to treat NSCLC. These include vincristine, fluorouracil, methotrexate, clotrimazole, etoposide, tamoxifen, sorafenib, doxorubicin, and pazopanib. These drugs are good at controlling genes, so the results of this study back them up.

Multiple studies have looked at how vincristine, a vinca alkaloid,^46^ and other common chemotherapeutic drugs can work together to treat NSCLC.^48^, ^49^, ^50^, ^51^ This alkaloid (the vincristine drug) acts as an anti‐microtubule agent that blocks mitosis by blocking cells in the metaphase.^52^

Fluorouracil is a chemotherapy drug that targets TYMS and has shown efficacy in treating NSCLC. 5‐Fluorouracil has been investigated for its anticancer properties, including against NSCLC.^53^, ^54^ It has been looked into methotrexate for the synergistic chemotherapy of NSCLC^55^ and is another chemotherapy drug that targets folate metabolism. Clotrimazole, which is commonly used as an antifungal medication, has shown potential as an antitumor drug in reducing the size and growth of neoplasms in previous studies.^56^ In a study carried out by Sebastian et al., a trend towards longer survival with concomitant clotrimazole and ICI for advanced NSCLC was observed.^57^ Etoposide is a topoisomerase II inhibitor that has been used in combination with other chemotherapy drugs for the treatment of NSCLC.^58^ Tamoxifen is a selective estrogen receptor modulator that has been investigated for its potential in treating breast cancer^59^ and is still under investigation for treating NSCLC. Sorafenib is a multi‐kinase inhibitor that targets several signaling pathways involved in cancer development and progression.^59^ Doxorubicin is an anthracycline antibiotic and one of the most commonly used chemotherapy drugs.^61^ Pazopanib is a tyrosine kinase inhibitor that targets angiogenesis and has been investigated as a potential treatment for cancer.^62^ These drugs hold promise as potential treatments for NSCLC and warrant further investigation.

Despite identifying potential drug candidates for NSCLC treatment, further investigation is necessary to evaluate their efficacy for specific cancer conditions. In‐silico studies could provide additional evidence to support the potential repurposing of these drugs. The findings of this study highlight the importance of gene co‐expression and network analysis in drug development for existing diseases and the advantages of drug repurposing as a quicker and more cost‐effective approach with potential improved safety profiles. The identified drugs in this study hold promise as effective treatments for NSCLC and warrant further investigation.

## 5 CONCLUSION

In this research paper, we propose a systems biology and network analysis approach to identify potential drugs for the treatment of non‐small cell lung cancer (NSCLC). We addressed the limitations of current treatments, such as drug resistance, toxicity, and low survival rates, by utilizing transcriptomics data and constructing gene co‐expression and co‐occurrence networks. Through our analysis, we identified differentially expressed genes in NSCLC and identified two gene co‐expression modules. By leveraging the Drug–Gene Interaction Database, we identified candidate drugs that target essential genes within these modules. Additionally, we constructed a drug–gene interaction network and validated the candidate drugs using gene set enrichment analysis.

Unlike previous research that solely relied on gene co‐expression networks, our approach considered both cancer cells and adjacent normal cells, thereby potentially reducing the side effects of treatment. The candidate drugs we identified, including topoisomerase inhibitors and proteasome inhibitors, have demonstrated effectiveness in preclinical and clinical studies for NSCLC and other cancers. Nevertheless, our study has several limitations. It relies on the assumption that the drug–gene interaction database is comprehensive and accurate, which may not always be the case. Additionally, transcriptomics data may not fully capture proteomics and metabolomics changes in NSCLC. Furthermore, being a computational analysis, our findings require further experimental validation.

## AUTHOR CONTRIBUTIONS

**Oluwatosin Maryam Adeyemo:** Conceptualization (lead); data curation (lead); formal analysis (lead); methodology (lead); supervision (supporting); writing – original draft (equal); writing – review and editing (supporting). **Zainab Ashimiyu‐Abdusalam:** Formal analysis (supporting); methodology (supporting); writing – original draft (equal). **Mary Adewunmi:** Supervision (lead); writing – original draft (equal); writing – review and editing (lead). **Temitope Ayanfunke Ayano:** Resources (equal); writing – original draft (equal). **Muhammad Sohaib:** Resources (equal); writing – original draft (equal). **Reem Abdel‐Salam:** Writing – original draft (equal).

## CONFLICT OF INTEREST STATEMENT

The authors have stated explicitly that there are no conflicts of interest in connection with this article.

### ETHICS STATEMENT

There are no ethical or financial issues, conflicts of interest, or animal experiments related to this research.

## Data Availability

Data sharing is not applicable to this article as no new data were created or analyzed in this study.
